# Listening to music during a repeated sprint test improves performance and psychophysiological responses in healthy and physically active male adults

**DOI:** 10.1186/s13102-023-00619-1

**Published:** 2023-02-21

**Authors:** Nidhal Jebabli, Abderraouf Ben Aabderrahman, Daniel Boullosa, Hamdi Chtourou, Nejmeddine Ouerghi, Fatma Rhibi, Karuppasamy Govindasamy, Ayoub Saeidi, Cain C. T. Clark, Urs Granacher, Hassane Zouhal

**Affiliations:** 1 Research Unit: Sport Sciences, Health and Movement, High Institute of Sport and Physical Education of Kef, UR22JS01, University of Jendouba, 7100 Kef, Tunisia; 2 Higher Institute of Sport and Physical Education of Ksar-Said, University of Manouba, Manouba, Tunisia; 3 INISA, Federal University of Mato Grosso do Sul, Campo Grande, Brazil; 4 Higher Institute of Sport and Physical Education of Sfax, University of Sfax, Sfax, Tunisia; 5 M2S (Laboratoire Mouvement, Sport, Santé) - EA 1274, Univ Rennes, 35000 Rennes, France; 6 Department of Physical Education and Sports Science, SRM Institute of Science and Technology, Kattankulathur, Tamilnadu India; 7 Department of Physical Education and Sport Sciences, Faculty of Humanities and Social Sciences, University of Kurdistan, Sanandaj, Kurdistan Iran; 8 Centre for Intelligent Healthcare, Coventry University, Coventry, UK; 9 Department of Sport and Sport Science, Exercise and Human Movement Science, University of Freiburg, Freiburg, Germany; 10 Institut International des Sciences du Sport (2IS), 35850 Irodouer, France

**Keywords:** Pacing strategy, Repeated sprint performance indices, Heart rate, Blood lactate concentration, Ratings of perceived exertion, Feeling scale

## Abstract

**Background:**

It is well-documented that listening to music has the potential to improve physical performance during intense physical exercise. Less information is available on the timing of music application. This study aimed to investigate the effects of listening to preferred music during the warm up of a subsequent test or during the test on performance of repeated sprint sets (RSS) in adult males.

**Methods:**

In a randomized cross-over design, 19 healthy males (age, 22.1 ± 1.2 years; body mass, 72.7 ± 9.3 kg; height, 1.79 ± 0.06 m; BMI, 22.6 ± 2.2 kg m^−2^) performed a test including 2 sets of 5*20-m repeated-sprints under one of three conditions: listening to preferred music during the test; listening to preferred music during the warm-up; or not listening to music. The assessed parameters comprised RSS performance indices, blood lactate, heart rate, the pacing strategy profile, rating of perceived exertion, and a feeling scale.

**Results:**

For performance indices during set 1 of the RSS test, we found a significant decrease in total sum sequence, fast time index and fatigue index in the listening to preferred music condition compared to the no music condition (total sum sequence: p = 0.006, d = 0.93; fast time index: p = 0.003, d = 0.67; fatigue index: p < 0.001; d = 1.30) and the listening to preferred music during the warm-up condition (fast time index: p = 0.002; d = 1.15; fatigue index: p = 0.006; d = 0.74). However, there was no significant effect of listening to preferred music on physical performance during set 2 of the RSS test. Compared to the no music condition, blood lactate concentrations were higher in the listening to preferred music during the test condition (p = 0.025; d = 0.92). In addition, listening to preferred music appears not to have an effect on heart rate, the pacing strategy profile, perceived exertion, and affective responses before, during and after the RSS test.

**Conclusion:**

Findings from this study revealed that RSS performances were better (FT and FI indices) in the PMDT compared with the PMWU condition. Moreover, in set 1 of the RSS test, better RSS indices were found in the PMDT compared to NM condition.

## Introduction

There is evidence indicating that athletes regulate their energy during competition in order to optimize performance [1–4]. This so-called pacing strategy refers to the variation of speed by regulating the rate of energy expenditure to prevent homeostatic disturbances during exercise or competition [5, 6]. When performing repeated sprint exercises, athletes may benefit from an ‘all-out’ strategy [2]. This profile consists of a fast beginning in the first sprint followed by a gradual subconscious performance decline up until the last sprint [2]. During competition, the interaction between psychophysiological stress components (e.g., rating of perceived exertion [RPE], anxiety, heart rate, lactate) may act as a central governor to regulate the pacing strategy by delaying the onset of fatigue and discomfort symptoms [7]. Previous studies have suggested that the pacing strategy could be influenced by other internal and external factors, such as the knowledge of the end point (knowledge of time or distance) [7], performance level [8, 9], the presence of other competitors [2], and listening to music [4, 10]. It seems that listening to music has the potential to modify the pacing strategy by positively affecting performance during physical exercise [10].

However, previous studies revealed controversial results regarding the effects of listening to music delivered during the warm-up of a short-term high-intensity exercise bout or during the actual exercise bout or performance test [11–24]. It has been postulated that listening to music during the warm-up [17–20] or during the exercise/test [21] has the potential to enhance high-intensity exercise performance. For example, Belkhir et al. [20] observed that listening to preferred music applied during the warm-up compared with neutral music has a positive effect on short-term maximal performance in soccer players, irrespective of the time of day. In contrast, other researchers reported no beneficial effects of listening to music on performance during high-intensity physical exercise [16, 22–24]. In fact, Ballmann et al. [16] reported that listening to preferred music did not positively affect repeated Wingate test performance in active males. Thus, based on these observations, it remains unclear as to whether listening to music has a stimulating effect on performance during high-intensity exercise. In addition, only limited knowledge exists on the effect of the timing of music application during physical exercise.

Accordingly, the aim of this study was to examine the effects of listening to preferred music on the pacing strategy, heart rate, perceived exertion, and feeling states during the performance of repeated sprints (RSS) in healthy male sport science students. Based on findings from previous studies, we hypothesized that listening to preferred music during the test is more effective than listening to music during the warm-up, and not listening to music at all on repeated sprint performance [17, 21].

## Materials and methods

### Participants

Nineteen healthy male sport-science students (age, 22.1 ± 1.2 years; body mass, 72.7 ± 9.3 kg; body height, 1.8 ± 0.1 m; BMI, 22.6 ± 2.2 kg m^−2^) volunteered to participate in this study. The enrolled sport science students practiced similar exercise volumes as part of their weekly training as sport science students (6 h wk^−1^). All participating individuals were experienced semi-professional soccer players (2.0 ± 0.5 years). Prior to the start of the study, all participants received a medical screening from a medical doctor. Based on the evaluation of the medical doctor, all participants were diagnosed as free from lower limbs musculoskeletal injuries that would affect test performance. All participants were informed on the procedures, potential risks, and benefits of the study. The study was approved by the local ethics committee of the Higher Institute of Sport and Physical Education of Ksar-Said, Tunis, Tunisia, and the protocol was carried out in accordance with the latest version of the Declaration of Helsinki.

### Study design

Five test sessions were scheduled with a rest period of at least 48 h between the sessions. During the first and second test session, anthropometric characteristics (body mass; body height; BMI) were taken from all participants and a familiarization session (sprint 20-m and RSS protocols) was conducted to verify RSS test-retest reliability. The experimental sessions were scheduled in randomized order. As such, the participants completed three experimental conditions: (1) listening to preferred music during the RSS test (PMDT), (2) listening to preferred music during the warm-up of the RSS test (PMWU), and (3) not listening to music (NM). Randomization was carried out with regards to the experimental conditions (i.e., preferred music during the test; preferred music during the warm-up; no music) using a freeware online tool (https://www.randomizer.org).

Before the RSS started, participants performed a standardized 10-min warm-up, including 4-min of light jogging (heart rate: 110–145 beat min^−1^), lateral displacements, dynamic stretching and jumping, followed by 3-min of passive recovery.

All tests were performed at the same time of day (± 1 h), to avoid any potential effect of diurnal variation on performance. All participants were asked to follow their normal diet during the time of the study.

The same investigator conducted the tests during all experimental sessions in a gymnasium at a comfortable temperature. Study participants and investigators were not blinded to the three experimental conditions.

### Listening to music

All participants selected their preferred songs. Using the “Edjing Mix” software (version 6.45.00, Android, France), to determine music tempo, we noticed that the selected music of the participants was characterized by a fast rhythm tempo (> 140 beats min^−1^). Therefore, regardless of the music selected, all songs had a fast tempo (> 140 beats min^−1^) with the same music volume (moderate music, 70 dB), monitored by a sound meter app (Sound Meter v1.5, Abc Apps, USA), and utilizing the same mp3 player Bluetooth Wireless Earphone.

### Test for the assessment of repeated sprint sets (RSS)

**Fig. 1:**
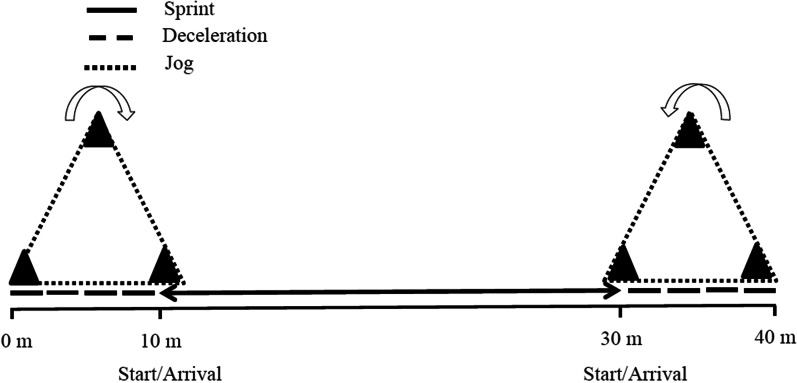
Multiple repeated sprint set test

The test for the assessment of RSS consisted of two sets of 5 × 20-m maximal sprints, with 15-s of active recovery (10-m jog) between the sprints, and another 1 min rest between sets. During the active recovery and between the sprints, participants were asked to slow down and complete 10-m in a jogging mode (Fig. 1) [27]. No verbal encouragement was provided during the RSS test. Through the assessment of linear sprint time, we were able to determine the total sum of sequences (TSS) which were defined as the total sprint time recorded during each set (5 sprints). In addition, we assessed the fastest sprint time (FT) which was defined as the fastest sprint time recorded during an individual set. Finally, we assessed the fatigue index (FI) which corresponded to the relative power decrement. FI was calculated according to the formula published by Fitzsimons et al. [28]:

$$\text{FI}\, (\%) = \text{TSS}/ ([\text{FT}*\text{number of sprints}]-1) *100.$$

Electronic timing gates were used to assess RSS performance (Globus, Microgate, Italy) with an accuracy of 0.01 s. Pilot data from 19 participants, collected during two familiarization sessions on two different test days, were used to determine the test-retest reliability of the RSS test.

### Blood lactate concentration

To measure blood lactate concentration, blood samples were taken from the fingertip (5 µl of blood) three minutes after the RSS test (Lactate Pro; Arkray, Tokyo, Japan).

### Heart rate

During the RSS test, peak and mean heart rate (HRpeak, HRmean) were recorded during the test using an individual heart rate monitor (Polar TF4, Polar Electro, Kempele, Finland).

### Ratings of perceived exertion and feeling scale

Before and after the RSS tests, the ratings of perceived exertion (RPE, 6–20) using the Borg scale [29] was obtained to estimate overall physical exertion. Additionally, before and after the RSS test, the feeling scale was used to measure the affective dimension (pleasure and displeasure) of the participants, ranging from − 5 (very bad) to + 5 (very good) [30].

### Statistical analyses

Normal data distribution was tested and confirmed using the Shapiro Wilk test. Accordingly, data were presented as means and standard deviations (SDs). Test-retest reliability was assessed for all variables using Cronbach’s model of the intraclass correlation coefficient (ICC) and the coefficient of variation (CV).

A one-way analysis of variance (ANOVA) was performed for HRpeak, HRmean, and blood lactate to discern between-condition differences at post. The outcome RSS indices (TSS; FT; FI) were analyzed using a three (conditions: listening to music during the warm-up, listening to music during the test, not listening to music) by two (sets: set 1, set 2) repeated measures ANOVA. Moreover, pacing strategy was evaluated using three conditions: (e.g., listening to music during the warm-up, listening to music during the test, not listening to music) by two sets (e.g., sprints in each set) repeated measures ANOVA. Additionally, data of RPE and FS were analyzed using a three (condition: listening to music during the warm-up, listening to music during the test, not listening to music) by two (time: pre, post) repeated measures ANOVA.

If condition-by-time or condition-by-set interactions turned out to be significant, Bonferroni adjusted post-hoc tests were used in the form of pair-wise comparisons. Partial eta squared (η^2^p) were taken from ANOVA output and used as effect sizes. Cohen’s d (d) were calculated to quantify meaningful differences in the data with demarcations of trivial (< 0.2), small (0.2–0.59), medium (0.60–1.19), large (1.2–1.99), and very large (≥ 2.0) [31]. Statistical significance was accepted, a priori, at p < 0.05. Data were analyzed using the SPSS 17 package (SPSS Inc., Chicago. IL).

## Results

According to the results from the Shapiro Wilk Test, data from all dependent variables were normally distributed. P-values ranged from 0.07 to 0.713.

### Test for the assessment of repeated sprint sets (RSS)

Data from the performance indices of the RSS test (TSS, FT, IF) are displayed in Table 1. For TSS, significant main effects of condition (F_(2, 54)_ = 4.22; p = 0.02; ηp^2^ = 0.12; power = 0.72) and set (F_(1, 54)_ = 69.23; p < 0.001; ηp^2^ = 0.52; power = 1) were found. However, the analysis indicated no significant condition-by-time interaction (F_(2,54)_ = 2.39; p = 0.1; ηp^2^ = 0.07; power = 0.46). Main effects of condition can be interpreted as a significant TSS decrease during PMDT (p = 0.006; d = 0.93) compared to NM during set 1. Moreover, no significant difference was observed between PMWU and NM (p = 0.331; d = 0.38) or between PMDT and PMWU (p = 0.065; d = 0.56).

**Table 1:** Comparisons of RSS outcomes, heat rate and lactate between PMDT, PMWU, and NM conditions (N = 19)

Variables	Sets	No music	Preferred music during test	Preferred music during warm-up	p (eta-squared [ηp^2^])		
					Main effect of condition	Main effect of time	Condition*time interaction
TSS (s)	Set1	17.84 ± 0.79	17.08 ± 0.81*	17.64 ± 0.72	0.02 (0.12)	< 0.001 (0.52)	0.1 (0.07)
	Set 2	18.25 ± 0.79	17.73 ± 0.80	18.01 ± 0.73			
FT (s)	Set 1	3.39 ± 0.19	3.28 ± 0.17*^$^	3.45 ± 0.15	0.06 (0.09)	< 0.001 (0.55)	< 0.001 (0.40)
	Set 2	3.53 ± 0.13	3.44± 0.16	3.47 ± 0.15			
FI (%)	Set 1	5.21 ± 2.62	1.62 ± 1.42*^$^	2.60 ± 2.17	< 0.001 (0.29)	0.061 (0.06)	0.001 (0.21)
	Set 2	4.03 ± 1.86	3.18 ± 1.54	3.98 ± 1.59			
HR mean (beat/min)		166 ± 10.68	171.2 ± 11.49	165.6 ± 8.16	0.13 (0.06)	--	--
HR max (beat/min)		178.7 ± 7.48	183.5 ± 10.54	180.4 ± 7.43	0.18 (0.05)	--	--
Lac post-test (mmol/L)		15.50 ± 1.34	16.82 ± 1.53*	16.18 ± 1.40	0.012 (0.45)	--	--

Data presented as mean (± SD), with ANOVA output reporting as P value (partial eta squared effect size)

*TSS* total sum sequence, *FT* fast time, *FI* fatigue index

*Significantly different between PMDT versus NM (p < 0.05); ^Ŧ^significantly different between PMDW versus NM (p < 0.05); ^$^significantly different between PMDT versus PMDW (p < 0.05)

For FT, no significant main effect of condition (F_(2, 54)_ = 2.94; p = 0.06; ηp^2^ = 0.09; power = 0.55) was found. However, a significant main effect of set (F_(1, 54)_ = 82.60; p < 0.001; ηp^2^ = 0.55; power = 1) was found as well as a significant condition-by-set interaction (F_(2, 54)_ = 17.10; p < 0.001; ηp^2^ = 0.40; power = 1). Bonferroni-adjusted post-hoc tests showed that in set 1 of the RSS test, FT was significantly lower during PMDT compared to NM (p = 0.003; d = 0.67) and PMWU (p = 0.002; d = 1.15). However, no significant between condition differences were detected during set 2.

For FI, results indicated a significant main effect of condition (F_(2, 54)_ = 13.04; p < 0.001; ηp^2^ = 0.29; power = 0.99), a significant main effect of set (F_(1, 54)_ = 24.35; p < 0.001, ηp^2^ = 0.40; power = 0.99), and a significant condition-by-set interaction (F_(2, 54)_ = 8.33; p = 0.001; ηp^2^ = 0.21; power = 0.96). Bonferroni-adjusted post-hoc tests revealed for set 1 of the RSS test lower values for PMDT compared to NM (p < 0.001; d = 1.30), PMWU compared to NM (p < 0.001; d = 1.28), and PMDT compared to PMWU (p = 0.006; d = 0.74). During set 2, no significant differences were found between the three conditions.

A pilot study was conducted to obtain reliability and sensitivity data for the applied parameters using 19 participants. The analysis showed an ICC of 0.93 (95% confidence interval [CI] 0.82–0.97) for TSS, an ICC of 0.96 (95% CI 0.91–0.98) for FT, and an ICC of 0.99 (95% CI 0.98–0.99) for FI.

In addition, we computed CVs across trials during the familiarization session. For TSS, the CV amounted to 1%, for FT to 1% and for FI to 11%.

### Heart rate frequency

The evaluation of HR frequency during the the RSS test is displayed in Table 1. Using one-way ANOVA, no significant differences were found for HR_max_ (F_(2, 54)_ = 1.78; p = 0.18; ηp^2^ = 0.05; power = 0.36) and HR_mean_ (F_(2, 54)_ = 2.1; p = 0.13; ηp^2^ = 0.06; power = 0.42).

### Pacing strategy

**Fig. 2:**
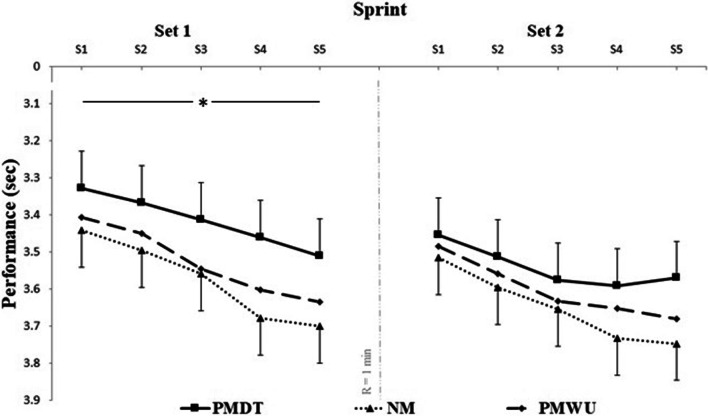
Performance for each sprint in RSS sessions. Data are presented as means and standard deviations. *PMDT* preferred music during test, *PMWU* preferred music during warm-up, *NM* no music, *R* recovery period; *Significant main effect of condition (p < 0.05)

Figure 2 details the effects of listening to preferred music on performances measured after each during the RSS test. Significant main effects of condition (F_(2, 54)_ = 5.72; p = 0.005; ηp^2^ = 0.15; power = 0.85) and set (F_(1, 54)_ = 45.1; p < 0.001; ηp^2^ = 0.70; power = 0.99) were observed for sprint performance. However, no significant condition-by- set F_(2, 54)_ = 0.84; p = 0.571; ηp^2^ = 0.05; power = 0.38) was found. Contrast analysis indicated a significant speed improvement for PMDT compared to PMWU (p = 0.019; d = 1.33) and NM (p = 0.002; d = 5.00).

In set 2, no significant main effects of condition (F_(2, 54)_ = 2.4; p = 0.098; ηp^2^ = 0.07; power = 0.47) and condition-by-set interaction (F_(2, 54)_ = 2.5; p = 0.552; ηp^2^ = 0.05; power = 0.39) were found. However, a significant main effect of set (F_(1, 54)_ = 12.50; p < 0.001; ηp^2^ = 0.77; power = 1) was observed.

### Blood lactate concentration

Data on blood lactate concentration are presented in Table 1. Using one-way ANOVA, blood lactate was significantly higher only in PMDT (p = 0.025; d = 0.92) compared to the NM condition. However, no significant difference was observed for blood lactate concentration between the PMDT and PMWU conditions.

### Ratings of perceived exertion and feeling scale

The RPE and the feeling state scores were recorded before and after the RSS test and are displayed in Table 2.

**Table 2:** Mean (SD) for rated perceived exertion (RPE) and feelings states scores recorded before and after the RSS (N = 19)

Variable	Time	No music	Preferred music during test	Preferred music during warm-up	p (eta-squared [ηp^2^])		
					Main effect of condition	Main effect of time	Condition*time interaction
RPE (AU)	Pre	12.41 ± 1.97	11.82 ± 2.22	11.73 ± 2.43	0.11 (0.06)	< 0.001 (0.81)	0.39 (0.02)
	Post	16.82 ± 1.53^¤^	15.5 ± 1.34^¤^	16.18 ± 1.40^¤^			
Feeling scale (AU)	Pre	2 ± 1.83	2.23 ± 2.27	2.136 ± 2.15	0.12 (0.06)	0.88 (0.003)	0.45 (0.03)
	Post	1.68 ± 1.17	2.82 ± 0.80	2 ± 1.27			

Data presented as mean (± SD), with ANOVA output reporting as p value (partial eta squared effect size)

*AU* arbitrary units

^¤^Significantly different between pre and post test for each condition (p < 0.05)

For RPE, a significant main effect of time (F_(1, 63)_ = 260.11; p < 0.001; ηp^2^ = 0.81; power = 1) was observed. However, no significant condition-by-time interaction (F_(2, 63)_ = 0.93; p = 0.39; ηp^2^ = 0.02; power = 0.20) was found.

For FS score, results indicated no significant main effect of condition (p = 0.12; ηp^2^ = 0.06), no significant main effect of time (F_(1, 63)_ = 0.02; p = 0.88; ηp^2^ = 0.003; power = 0.05), and no significant condition-by-time interaction (F_(2, 63)_ = 0.8; p = 0.45; ηp^2^ = 0.03; power = 0.18).

## Discussion

To our knowledge, this is the first study that investigated the effects of the moment of listening to preferred music on pacing strategy, HR frequency, perceived exertion, and feeling state during sequences of RSS tests. In agreement with our research hypothesis, the main finding of the present study revealed that FT and FI indices, during RSS, were higher with PMDT compared with the PMWU condition. Moreover, in set 1 of the RSS test, better indices were found for PMDT compared to NM.

In contrast, no significant effect of listening to music was observed for the pacing strategy profile, HR, RPE and feeling scale.

This study showed better RSS performance (TSS, FT, FI) in set 1 for the PMDT compared to the NM condition. These differences between conditions could only be found in set 1 but not in set 2. In agreement with the results of set 2, and in disagreement with results of set 1, previous studies have shown no effects of listening to music on repeated sprint performances during different experimental conditions [24, 33]. Indeed, Atan [24] observed no significant difference of fast or slow music on repeated sprint performance (RAST) of 6 * 35-m with 10 s of recovery between tests. Also, Eliakim et al. [32] showed that music did not affect the performance (total time; IF) of 12 * 20-m sprint on young male basketball players. However, Rhoads et al. [14] reported that listening to preferred music during repeated Wingate test improve power output in young males’ athletes. The discrepancies between these findings and our results might be due to the difference in the choice of music type (preferred, not preferred, synchronized, non-synchronized), tempo (slow, fast) participants' fitness levels (trained, untrained), and test procedure (sprint number).

In fact, Brownley et al. [33] previously confirmed that the music elicited a greater mean peak power in untrained subjects compared to the trained subjects during low and high-intensity exercises. In addition, all the preferred music sequences were of high tempo (> 140 bpm) which might be one of the factors that improved the performance indices. Moreover, previous studies have shown that listening to fast tempo music during high intensity exercise increases salivary cortisol concentration which improves substrate availability during exercise and recovery though increased gluconeogenesis and free fatty acid mobilization [26, 34]. In agreement with the observed decrease in the FI index during PMDT and PMWU, different studies reported that listening to music can promote systemic physiological changes during exercise by increasing brain activity in the left inferior frontal gyrus and regions of the insular cortex. In line with the central governor theory, listening to music may lead to an attenuation of internal fatigue-related signals and thus improved physical performance [9, 26]. In addition, Sugiharto et al. [35] observed that listening to music during exercise improves irisin levels after high-intensity exercise in young non-professional athletes. This enhancement may directly contribute to the gradual alteration of energy expenditure. In the current study, there was no significant difference of HR_mean_ and HR_max_ observed during the two experimental sessions. These findings suggest that the preferred music used as an external stimulus could not influence the heart responses, and probably cardiorespiratory responses, during glycolytic exercise.

Furthermore, participants were able to improve performance during PMDT session without change on cardiovascular activity compared to NM session. In agreement with the present study, Atan, [24] showed that listening to music during RAST did not significantly affect HR responses. In another study, HR was not affected between the four conditions (fast upbeat music, classical music, preferred music, and no music conditions) during similar exercise intensity [36]. Moreover, Nakamura et al. [25] reported that, during high-intensity exercise, music was unable to influence the physiological response, irrespective of music type. However, our results contrast with the previous reports that showed that the HR was significantly changed, during warm-up and during the test in the music condition [17, 32], probably due to the difference in the choice of the study population and exercise protocols. To our knowledge, this is the first study to demonstrate the effect of preferred music during a RSS protocol on pacing strategy. During sets 1 and 2 of the RSS protocol, the data supported the existence of a pacing strategy profile that is stable and unaffected by the effect of listening to preferred music. Indeed, our results confirm that participants exhibited the same decreasing-profile strategy (“all out” strategy) in the three conditions and the two sets of the RSS test. This strategy has been found in previous studies as well [36, 38]. Keller [37] reported that an all-out pacing strategy is optimal in running races with a distance up to 291 m. In addition, Gastin [38] was able to show that the contribution of the anaerobic metabolism became significantly less with maximal exercise tests of 30 to 60 s duration. This is mainly due to reduced ATP turnover which seems to be partially compensated by an increase in oxygen uptake during the following sprints. However, the results of the present study showed that the observed decrement in speed during RSS, was different in PMDT compared with NM, especially during set 1. These findings may be due to the adoption of a muscle recruitment strategy that ensures maximal work during exercise, particularly during the PMDT condition, without biological failure [38].

According to previously published studies, the effect of listening to music on physical performance is related to psychophysiological factors which lead to an increase activation in the motor cortex and autonomic response [16, 26]. With increasing RSS performance, blood lactate levels after exercise were significantly higher in PMDT compared to NM. The specific reasons for this result remain largely unknown. A previous study [26] has shown that listening to music during exercise may have a relaxing effect. With less muscular tension, there could be increased blood flow and therefore greater lactate clearance [26]. In this context, we hypothesized that the improvement in RSS indices, during the PMDT, may indicate that the participants have inhibited the activity of the glycolysis thus leading to a larger lactate accumulation and increased cellular acidosis.

However, this mechanism remains largely speculative. Interestingly, Atan [24] reported no significant difference in blood lactate after sprint and Wingate tests with or without music in active male participants.

In the present study, there were no significant condition-by-time interactions for RPE. Our results are in line with findings from Eliakim et al. [32], who showed that listening to preferred music did not affect RPE before or after a repeated sprint test. This can most likely be explained by several factors, including the expertise status of the participants (trained, untrained), the exercise load (pacing strategy profiles) and the time point of RPE testing (before, during and after exercise). Karageorghis and Priest [39] showed that motivational effects of listening to music appear to be related to increased individual perceptions of self-esteem and sense of confidence. An increase in exercise intensity of work during the listening to music condition elicits a higher RPE response compared to the no music condition [40].

Psychological responses, along with listening to music, can influence physical performance as they relate to well-being, emotional, behavioral and cognitive domains [33, 41]. Indeed, emotion and motivation may play a relational role with cortical treatment and visceral information on the behavioral and physiological state of the subject [41]. Our results revealed no significant condition * time interaction; although, other studies, like Elliott et al. [42] stated that listening to motivational music favored a positive emotional reaction associated to the improvement of total distance covered during 20 min cycle ergometer trials. It has been also suggested that a greater activation of glycolytic metabolism could not influence feeling responses [26]. Furthermore, these findings could be attributed to the possible role of subject’s experience with music during their formative years. According to Rejeski [43], the affectivity of music, as an external cue, is limited with the appearance of internal cues as fatigue, and their effect on mental status during high-intensity exercise. The interaction between psychological and physiological parameters are closely linked to the ergogenic effect of music [16]. However, the mechanism by which music preferentially allows better performance is still not well understood [26]. As such, the current study has some limitations that should be acknowledged. More research is needed to elucidate the interactions between psychological and physiological parameters while listening to preferred music during high-intensity interval training.

## Conclusion

In summary, the findings of this study showed that listening to music applied during the RSS test compared to the no music condition was more effective to improve RSS performance. This is substantiated through the observed decrease in total sum sequence, fast time index and fatigue index.

Coaches and athletes are advised to listen to preferred music during tests/exercises and not just during the warm-up to improve their sprint performance.

## Data Availability

The datasets generated during and analyzed during the current study are not publicly available due to confidential information about the participants but are available from the corresponding author on reasonable request.

## Abbreviations

PMDT: Listening to preferred music during the test
PMWU: Listening to preferred music during the warm-up
NM: Not listening to music
RPE: Rating of perceived exertion
FS: Feeling scale
